# High expression of IDO1 and TGF-β1 during recurrence and post infection clearance with *Chlamydia trachomatis*, are independent of host IFN-γ response

**DOI:** 10.1186/s12879-019-3843-4

**Published:** 2019-03-04

**Authors:** Noa Ziklo, Wilhelmina M. Huston, Kuong Taing, Peter Timms

**Affiliations:** 1 Faculty of Science, Health, Education & Engineering, University of the Sunshine Coast, Sippy Downs, Sunshine Coast, QLD Australia; 2 School of Life Sciences, Faculty of Science, University of Technology, Sydney, Australia; 3 Sunshine Coast Sexual Health and HIV Service (Clinic 87), Nambour, Sunshine Coast, QLD Australia

**Keywords:** *Chlamydia trachomatis*, Indoleamine 2,3-dioxygenase, Interferon-gamma, Tryptophan, Kynurenine, Regulatory immune response

## Abstract

**Background:**

*Chlamydia trachomatis* infections in women continue to be a major public health concern due to their high prevalence and consequent reproductive morbidities. While antibiotics are usually efficient to clear the *Chlamydia*, repeat infections are common and may contribute to pathological outcomes. Interferon-gamma (IFN-γ)-mediated immunity has been suggested to be protective against reinfection, and represent an important anti-chlamydial agent, primarily via the induction of indoleamine-2,3 dioxygenase 1 (IDO1) enzyme. IDO1 catalyzes the degradation of tryptophan, which can eliminate *C. trachomatis* infection in vitro. Here, we sought to measure IDO1 expression levels and related immune markers during different *C. trachomatis* infection statuses (repeated vs single infection vs post antibiotic treatment), in vitro and in vivo.

**Methods:**

In this study, we measured the expression levels of IDO1 and immune regulatory markers, transforming growth factor β1 (TGF-β1) and forkhead box P3 (FoxP3), in vaginal swab samples of *C. trachomatis*-infected women, with either single or repeated infection. In addition, we used an in vitro co-culture model of endometrial carcinoma cell-line and peripheral blood mononuclear cells (PBMCs) to measure the same immune markers.

**Results:**

We found that in women with repeated *C. trachomatis* infections vaginal IDO1 and TGF-β1 expression levels were significantly increased. Whereas, women who cleared their infection post antibiotic treatment, had increased levels of IDO1 and TGF-β1, as well as FoxP3. Similarly, using the in vitro model, we found significant upregulation of IDO1 and TGF-β1 levels in the co-culture infected with *C. trachomatis*. Furthermore, we found that in PBMCs infected with *C. trachomatis* there was a significant upregulation in IDO1 levels, which was independent of IFN-γ. In fact, *C. trachomatis* infection in PBMCs failed to induce IFN-γ levels in comparison to the uninfected culture.

**Conclusions:**

Our data provide evidence for a regulatory immune response comprised of IDO1, TGF-β1 and FoxP3 in women post antibiotic treatment. In this study, we demonstrated a significant increase in IDO1 expression levels in response to *C. trachomatis* infection, both in vivo and in vitro, without elevated IFN-γ levels. This study implicates IDO1 and TGF-β1 as part of the immune response to repeated *C. trachomatis* infections, independently of IFN-γ.

**Electronic supplementary material:**

The online version of this article (10.1186/s12879-019-3843-4) contains supplementary material, which is available to authorized users.

## Background

Sexually transmitted *Chlamydia trachomatis* infections continue to be a major public health concern due to high infection rates and long-term reproductive morbidities [1, 2]. While antibiotic treatment is generally efficient in clearing the infection [3], treatment failure and reoccurring infections are still common [4]. Protective immunity against *C. trachomatis* reinfections has been associated with interferon gamma (IFN-γ) production in several human studies [5–7]. Various in vitro studies with *C. trachomatis*-infected cell lines have also shown the important role of IFN-γ in eliminating infection [8, 9], due to the induction of the enzyme indoleamine 2,3-dioxygenase 1 (IDO1) [10, 11]. IDO1 is responsible for the catabolism of tryptophan to kynurenine [12]. Depriving this essential amino acid from the tryptophan auxotroph *C. trachomatis* have an inhibitory effect on infection in vitro. While high IFN-γ levels were shown to eradicate the infection, lower levels can drive urogenital *C. trachomatis* to enter their persistence form, characterized by in vitro aberrant, non-infectious bodies [13]. *C. trachomatis* aberrant bodies were also identified in infected women, and were associated with low IFN-γ levels in the genital tract [14]. Furthermore, low vaginal tryptophan levels were shown to be associated with spontaneous resolution of *C. trachomatis* infections in women [15]. Although chlamydial death due to tryptophan depletion via IFN-γ-induced IDO1 axis, has been well characterized in vitro, relatively few studies have measured IDO1 expression levels and its enzymatic activity in the actual infection site [16]. In a recent study, IFN-γ, tryptophan and kynurenine levels were measured in vaginal secretions of women who were infected with *C. trachomatis* (single or repeated infection) versus uninfected women [17]. It was reported that higher kynurenine to tryptophan ratios (kyn/trp) were associated with current, single or repeated *C. trachomatis* infections. Specifically, it was found that women with repeated *C. trachomatis* infection had significantly higher kynurenine levels in their vaginal secretions. High kyn/trp ratios however, did not correlate with the low IFN-γ levels measured from vaginal secretions of the same women. IDO1-IFN-γ axis, which is a well-known important antimicrobial mechanism [18, 19], is also responsible for downregulation of the pro-inflammatory response in the host [24–30]. The depletion of tryptophan, which is necessary for the survival and proliferation of T cells, causes their arrest in the G1 phase of the cell cycle [25]. This is partly due to the induction of stress response triggered by GCN2, a stress kinase that is activated by the elevation in uncharged tRNAs [24]. Tryptophan depletion can also inhibit T cell proliferation through inactivation of the mTOR pathway [23]. Another mechanism is through tryptophan catabolites, such as 3-hydroxyanthranilic and quinolinic acids that can induce T-cell apoptosis [22, 27]. Other kynurenine derivatives and kynurenine itself can induce the differentiation of naïve T cells to regulatory T cells (Tregs) [21], through the activation of the AhR [20]. IDO1 is inducible in different immune cells as well as mucosal epithelial cells, and is regulated by a complex immune signal cascade [19]. Aside from IFN-γ stimulus, there are other cytokines which induce IDO1 expression, such as by IFN-α, IFN-β, TGF-β, TNF-α and IL-1β [28–30]. In addition, lipopolysaccharide (LPS), extracted from cell membranes of gram-negative bacteria, was shown to induce IDO1 expression in IFN-γ-KO mice [31]. The LPS-stimulated IDO1-induction was shown to be largely dependent on TNF-α. Furthermore, IDO1 can act as a signal transducer that contributes to long-term tolerogenic phenotype of plasmacytoid dendritic cells (pDCs) in response to the immune-regulatory cytokine transforming growth factor β (TGF-β) [29]. TGF-β-conditioned pDCs induced forkhead box P3 (FoxP3), which is an essential transcription factor for the immuno-suppressive function of Tregs [32], whereas IFN-γ conditioning induced T cell apoptosis and suppressed proliferation. These data have led us to hypothesize that low IFN-γ levels in repeatedly infected women, that had high kynurenine levels [17], may be due to downregulation of the cytokine by the immune-regulatory properties of IDO1, as a result of recurrent chlamydial infections. *C. trachomatis* was shown to have several immune evasion mechanisms that allow it to establish infection, which can often persist for long periods of time without any symptoms [33]. These mechanisms include reduced inflammatory responses elicited by the chlamydial LPS, reduced adaptive immune response and enhanced survival inside the host cell (reviewed in [34]). Here, we have focused on a selection of immune genes, IDO1, IFN-γ, TGF-β1 and FoxP3, to examine the regulatory immune response during *C. trachomatis* single versus repeated infection and post antibiotic treatment, in a cohort of a previously published study [17]. In addition, the expression levels of these genes were measured in vitro using a *C. trachomatis* infected endometrium cell line (ECC1) co-cultured with PBMCs, with or without azithromycin treatment. We found that in women with repeated infections and in women who have cleared their infection after antibiotic treatment, immune-regulatory markers, IDO1, TGF-β1 and FoxP3 were significantly upregulated in comparison to *C. trachomatis* negative women and in those with single current infection. We also show that IDO1 induction was not accompanied by elevated IFN-γ levels. Although IFN-γ-induced IDO1 activity and tryptophan depletion was previously proposed to be an anti-chlamydial mechanism, our data suggest that repeated *C. trachomatis* infections, as well as antibiotic treatment, can induce IDO1 in an IFN-γ independent manner, that contribute to downregulation of the immune response. These mechanisms may assist the pathogen to avoid immunity and establish an infection.

## Methods

### Study cohort

In a previous published study [17], thirty-seven samples were collected from 25 women aged ≥19, who attended the Sexual Health Clinic in Nambour, Australia. Women were diagnosed for *C. trachomatis* infection using Cobas® 4800 CT/NG Test (Roche, Australia) (Rockett et al., 2010), and were either, *C. trachomatis* negative (CT-N; *n* = 10), *C. trachomatis* positive with single infection (CT-P; *n* = 11) or *C. trachomatis* positive with a recent repeated infection (up to 1 year) (CT-RP; *n* = 3). Other sexually transmitted infections were excluded from this study. Women with single *C. trachomatis* infection were treated with a single dose of azithromycin and were invited back to second and third visits for follow up (“post antibiotic treatment”- PAT; *n* = 13) (participants information can be found in Additional file 1 and Additional file 2). High vaginal swab samples and cervical secretion samples were collected from each woman. This study was approved by The University of the Sunshine Coast, Human Research Ethics Committee (number A/14/623), and The Prince Charles Hospital Human Research Ethics Committee (number HREC/14/QPCH/14). All participants provided written informed consent.

### Female human PBMCs processing

Human PBMCs were received from a 61 years old (*C. trachomatis* negative), Caucasian female donor, (Lonza, Australia, catalog number CC-2702, lot number 0000528306). Cryopreserved PBMCs were shipped in liquid nitrogen. Upon arrival, cells were placed immediately in liquid nitrogen for one week before use. PBMCs were quickly thawed in 37 °C water bath for 2 min, and were suspended in RPMI-1640 medium (Sigma-Aldrich, Australia), containing 10% heat inactivated fetal calf serum (FCS) (Life Technologies, Australia), treated with 20 U/ml DNase I (Qiagen, Australia). Cells were then left to rest for 12 h prior to the experimental assay [35]. Cell’s count was 5 × 10^7^ cell/vial, using hemocytometer.

### Cell culture conditions

*C. trachomatis* serotype D isolate (ATCC VR-885) was routinely cultured in ECC1 cell line (ATCC CRL-2923) with DMEM (Gibco, Australia) containing 5% heat inactivated fetal calf serum (FCS) (Life Technologies, Australia), 120 μg/ml streptomycin (Sigma-Aldrich, Australia), 50 μg/ml Gentamycin (Gibco, Australia), 37 °C, 5% CO_2_. ECC1 is an endometrial primary cell line, responsive to hormones and to *C. trachomatis* infections, was previously used in infectivity assays and similar co-culture models [36, 37]. Experiments were conducted in 48-well plates, where co-culture of 40,000 ECC1 cells/well and 8 × 10^5^ female human PBMCs (Lonza, Australia) cells/well were seeded with RPMI-1640 medium (Sigma-Aldrich, Australia) containing 10% heat inactivated FCS (Life Technologies, Australia), 120 μg/ml streptomycin (Sigma-Aldrich, Australia), 50 μg/ml Gentamycin (Gibco, Australia), 37 °C, 5% CO_2_. In addition, control plates with either ECC1 cells alone or human PBMCs alone were used. Female PBMCs donor was reported negative for *C. trachomatis*. Co-cultures were infected with *C. trachomatis* at a multiplicity of infection (MOI) of 0.1. At the time of infection, the ECC1 monolayer was at 90% confluence. At 20 h PI infected cells were treated with 1 μg/ml of azithromycin (Sigma-Aldrich, Australia). For evaluation of transcript levels, cells were harvested at 20 h post seeding, 44 h and 68 h PI. Infectious yields were measured at 44 h PI. Infected cells and culture supernatants were then sonicated and used to infect a new ECC1 cell monolayer in three replicates, for enumeration of recoverable inclusion forming units (IFUs). After staining with an in-house anti-HtrA and goat anti rabbit IgG (H + L) Alexa Flour 488 (Invitrogen, Australia), wells were visualized for inclusion presence using fluorescence microscopy (Nikon Eclipse TiS Fluorescent Microscope). The chlamydial-HtrA is a virulence and stress response serine protease and molecular chaperone, which is specific for unfolded proteins [38, 39]. The IFU/ml were determined for each condition by measuring the number of inclusions in multiple wells, taking into account the dilution and volume from the original culture. The detection limit of the assay when only one inclusion is visible via microscopy is 80 IFU/ml, according to the numbers of fields of view in the microscope. Infected or uninfected ECC1 cells and co-cultures of ECC1 and PBMCs were fixed at 36 h PI and were stained with Chlamydia LPS (Cellabs, Australia) to evaluate the ECC1 cell’s conditions and the presence of inclusions (Additional file 3).

### RNA purification and reverse transcription

High vaginal swab samples were stored in 1 ml of RNA*later* (Ambion, Australia) in − 80 °C until processed. Total RNA was extracted from the swabs using RNeasy micro kit (Qiagen, Australia), according to the manufacturer’s instructions. The RNA concentration and purity were determined using Nano-drop Spectrophotometer. 0.2 μg of total RNA was reverse transcribed using QuantiTect Reverse transcription kit (Qiagen, Australia), in accordance with the manufacturer’s instructions. For cell culture samples, ECC1, PBMCs and cell’s supernatant were transferred into 1.7 ml Eppendorf tube and centrifuged at 200×g/10 min/4 °C. Cells were resuspended in RNA*later* and stored in − 80 °C until processed. Total RNA was extracted from the culture samples using RNeasy mini kit (Qiagen, Australia), according to the manufacturer’s instructions. Prior to the reverse transcription, an additional DNase I step was performed. 0.2 μg of total RNA was reverse transcribed using QuantiTect Reverse transcription kit (Qiagen, Australia), in accordance with the manufacturer’s instructions.

### mRNA gene expression

Amplification was carried out according to the manufacturer’s instructions using QuntiNova SYBR Green PCR kit (Qiagen, Australia). The cycling program was 95 °C for 2 min followed by 40 cycles of 5 s at 95 °C and 10 s at 60 °C. Transcript levels were quantified using Rotor-GeneQ (Qiagen, Australia). Each primer set was optimized according to a standard curve, while RNA samples were used to assure no DNA contamination. For each sample, transcripts results were normalized against the mRNA of the housekeeping gene β-actin transcripts. The primers used for transcripts quantification are listed in Table 1. Results are presented as normalized values of 2^-ΔCT^.

**Table 1:** Primers’ sequences used for RT-qPCR

Gene	Primer sequence
β-actin	F: 5′-TACCTCATGAAGATCCTCA-3’
	R: 5′- TTCGTGGATGCCACAGGAC − 3’
IDO1	F: 5′-AGAAGTGGGCTTTGCTCTGC-3’
	R: 5′-TGGCAAGACCTTACGGACATCTC-3’
IFN-γ	F: 5′-ATTCGGTAACTGACTTGAATGTCC-3’
	R: 5′-CTCTTCGACCTCGAAACAGC-3’
TGF-β1	F: 5′- CCCAGCATCTGCAAAGCTC − 3’
	R: 5′-GTCAATGTACAGCTGCCGCA-3’
FoxP3	F: 5′-GCACCTTCCCAAATCCCAGT-3’
	R: 5′-GGCCACTTGCAGACACCAT-3’

### Statistical analysis

**Fig. 1:**
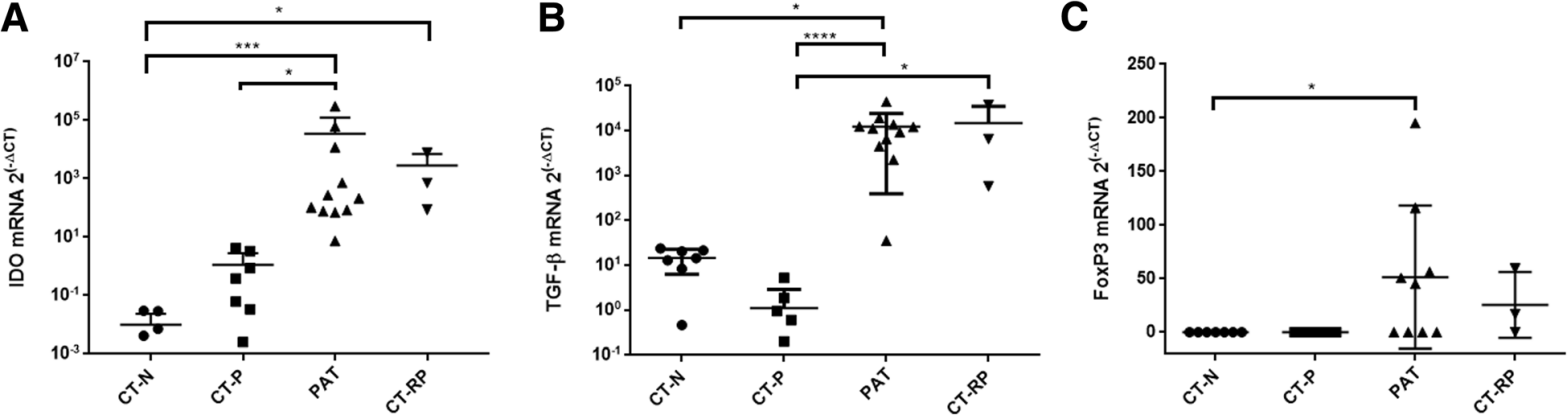
IDO1, TGF-β1 and FoxP3 mRNA expression levels in vaginal swab samples according to chlamydial infection status. Expression levels of the genes (**a**) IDO1, (**b**) TGF-β1 and (**c**) FoxP3 were measured from vaginal swab samples of women who were *Chlamydia* negative (CT-N; *n* = 7), *Chlamydia* positive (CT-P; *n* = 8), post antibiotic treatment (PAT; *n* = 11) and repeated *Chlamydia* infections (CT-RP; *n* = 3). Data are presented as mean ± SD of 2^(−ΔCT)^. Significant differences are presented in the graph (*p* < 0.05), using Kruskal-Wallis with Dunn’s post-test

**Fig. 2:**
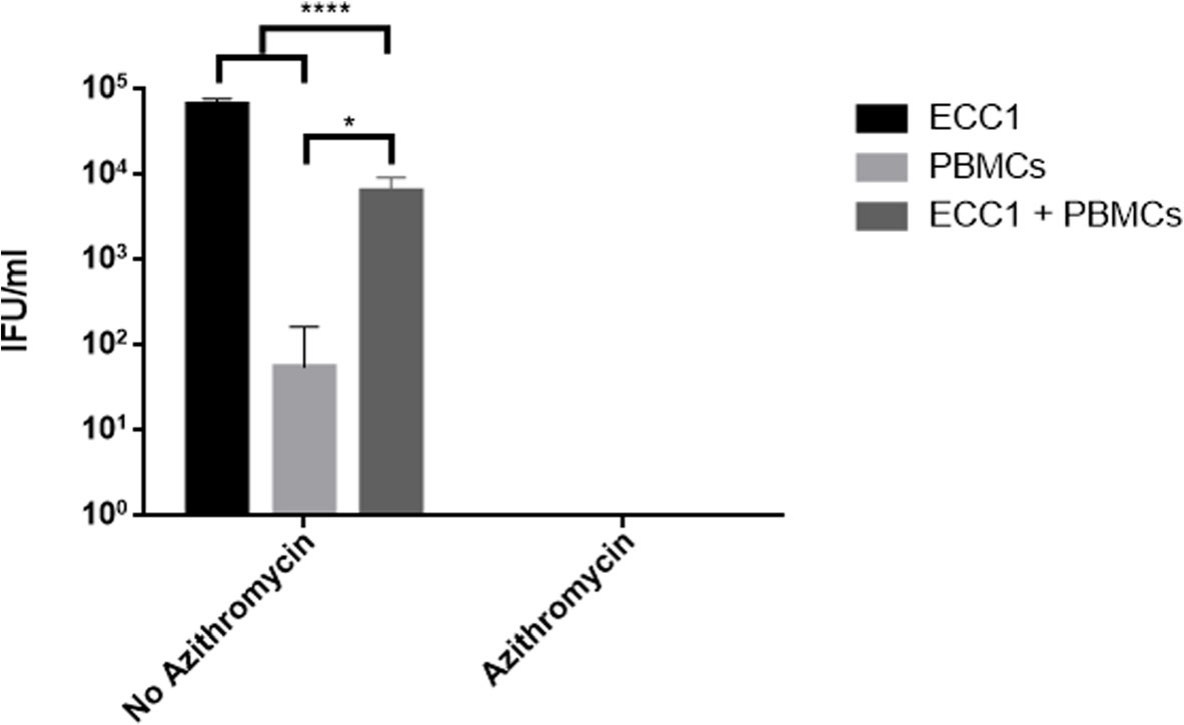
*C. trachomatis* infectivity from a cell culture of ECC1, human PBMCs and a co-culture of ECC1 and PBMCs, with or without azithromycin treatment. ECC1/PBMCs/co-culture of ECC1 and PBMCs were infected with *C. trachomatis* in MOI of 0.1. Cultures were either treated with azithromycin at 20 h PI, or not. The *Chlamydia* infected cells and culture supernatant were harvested a 44 h PI, sonicated and used to infect a new ECC1 monolayer for enumeration of recoverable IFUs. Data are presented as mean ± SD IFU/m (*n* = 9) determinations

**Fig. 3:**
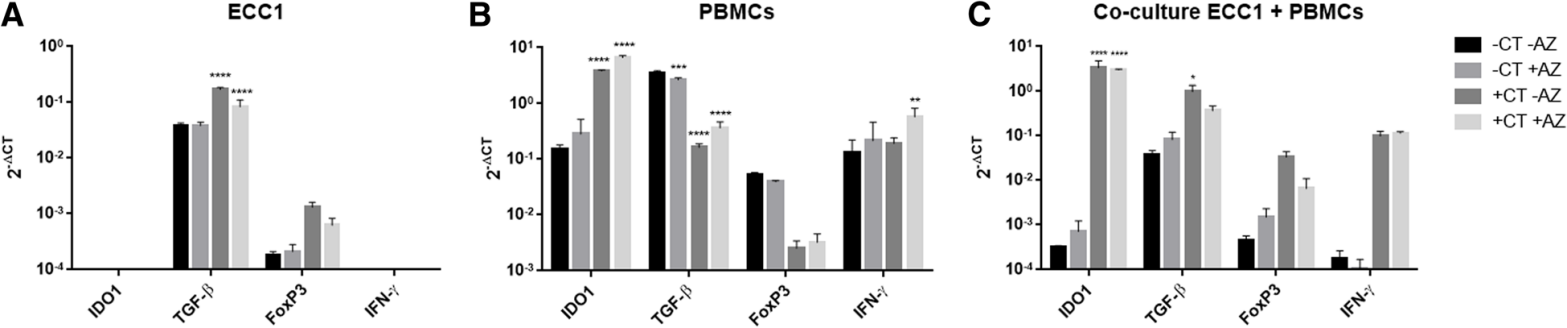
IDO1, TGF-β1, FoxP3 and IFN-γ expression levels as a response to *C. trachomatis* infection, with or without azithromycin treatment. Transcript levels of IDO1, TGF-β1, FoxP3 and IFN-γ (2^- ΔCT^) were measured from cell cultures of (**a**) human endometrial cell line ECC1, (**b**) PBMCs of *C. trachomatis* negative female, and (**c**) co-culture of ECC1 and PBMCs. Transcript levels were compared between treatments; without *Chlamydia* infection or azithromycin treatment (−CT –AZ), no *Chlamydia* infection with azithromycin (−CT + AZ), with *Chlamydia* infection no azithromycin (+CT –AZ) and with *Chlamydia* infection and azithromycin treatment (+CT + AZ). Cells were infected with *C. trachomatis* at MOI of 0.1. Azithromycin was added to the culture at 20 h PI, or at 44 h post seeding in control treatments. Total RNA was isolated from cultures at 44 h PI. Data are presented as mean ± SD. Significant differences are indicated in the graph (*p* < 0.05), using 2-way ANOVA with Dunnett’s multiple comparison post-test

All cell culture experiments were conducted in triplicate. The IFU/ml was determined for each condition by measuring the number of inclusions in multiple wells, and accounting for the dilution and volume from the original culture. Data were analyzed using Graph Pad Prism V. 7.01 (Graph Pad Software, Australia) and presented as the mean ± standard deviation (SD). Statistical differences were determined using Kruskal-Wallis, with Dunn’s post-test (Fig. 1), and two-way ANOVA for the in vitro experiments, with Tukey’s multiple comparison as a post-test in Fig. 2, and Dunnett’s multiple comparison test to the control treatment in Fig. 3.

## Results

### Differential expression levels of IDO1 and immune regulatory markers TGF-β1 and FoxP3 during *C. trachomatis* single versus repeated infection and post antibiotic treatment

The potential tryptophan degradation to kynurenine pathway during *C. trachomatis* infections in women with single or repeated infection was evaluated by the expression levels of IDO1 in swab samples. We found that in women with repeated *C. trachomatis* infection (CT-RP), IDO1 expression levels were significantly higher in comparison to *C. trachomatis* negative women (CT-N) (*p* < 0.05) (Fig. 1, a). Although not significant, *C. trachomatis* positive patients with single infection (CT-P) had a trend of higher IDO1 expression levels in comparison to *C. trachomatis* negative patients. This suggests that although initial *C. trachomatis* infection does induce IDO1 expression, repeatedly infected women exhibit a stronger IDO1 response in the vagina. Surprisingly, we found that in women that had a single infection (CT-P), after antibiotic treatment and infection clearance (PAT), IDO1 expression levels increased significantly (*p* = 0.0148) (Fig. 1, a).

In order to test for regulation of the immune response, we measured the expression levels of immuno-regulatory markers TGF-β1 and FoxP3 in the same women. Similar to IDO1 expression levels response, we found that TGF-β1 was significantly upregulated in women with repeated *C. trachomatis* infection, as well as in women post-antibiotic treatment, in comparison to *C. trachomatis* positive women with single infection (*p* < 0.0209 and *p* < 0.0001 respectively). When compared to *C. trachomatis* negative women, post antibiotic treatment group had significantly higher TGF-β1 levels (*p* = 0.0374), while repeatedly infected women had a trend of higher TGF-β1 although not significant (Fig. 1, b). On the other hand, this time, women with a single *C*. *trachomatis* infection had decreased TGF-β1 expression levels in comparison to *C. trachomatis* negative women (not significant). The regulatory transcription factor FoxP3 expression levels were significantly higher only in women post antibiotic treatment, in comparison to *C. trachomatis* negative women (*p* < 0.05) (Fig. 1, C). These results suggest that there is an apparent regulation of the immune response during repeated *C. trachomatis* infection, indicated by TGF-β1 and IDO1, and in particularly, post antibiotic treatment and infection clearance, indicated by IDO1, TGF-β1 and FoxP3. In addition, IDO1 was differentially expressed during different *C. trachomatis* infection statuses, with the highest levels observed in women post antibiotic treatment and during repeated infection. Longitudinal increase in IDO1 and TGF-β1 levels of individual women post antibiotic treatment and infection clearance can be found in Additional file 4.

### *C. trachomatis* inoculation in human PBMCs resulted in reduced infectivity in vitro

In order to test the host cell immune regulatory response to *C. trachomatis* infections and the effect of azithromycin treatment, we developed an in vitro model. Human endometrial carcinoma cell line (ECC1) was co-cultured with PBMCs cells sourced from a *C. trachomatis* negative female human. In addition, ECC1 and PBMCs were cultured separately as a comparison. Cells were infected with *C. trachomatis* and treated with 1 μg/ml of azithromycin at 20 h PI in order to clear the infection, as well as to evaluate whether azithromycin might have an effect on the increased expression levels of the immune regulatory genes IDO1, TGF-β1 and FoxP3, observed in women post antibiotic treatment. Cultures were then sonicated and used to infect a new ECC1 monolayer, to test for recoverable *C. trachomatis* IFUs. No chlamydial inclusions were found in any of the cultures treated with azithromycin (either ECC1, PBMCs or ECC1 and PBMCs co-culture) (Fig. 2)**.** In addition, infection of human PBMCs alone with live *C. trachomatis*, without the azithromycin treatment, resulted in significantly reduced, yet apparent infectivity (*p* < 0.0001). The number of chlamydial IFUs measured from ECC1 and PBMCs co-culture was significantly reduced in comparison to infected ECC1 culture alone (*p* < 0.0001) by almost two logs.

### Upregulation of IDO1 and TGF-β1 expression levels upon *C. trachomatis* infection in a co-culture of ECC1 cell line and PBMCs

We found that in *C. trachomatis* infected co-culture of ECC1 and PBMCs, IDO1 and TGF-β1 expression levels were significantly upregulated (*p* < 0.0001 and *p* < 0.05 respectively), when compared to uninfected cultures (Fig. 3, c). Similar to the co-culture, in PBMCs alone, IDO1 expression levels were significantly upregulated in response to *C. trachomatis* infection (*p* < 0.0001). However, TGF-β1 transcripts in PBMCs alone were initially high in the absence of infection*,* and were significantly downregulated upon infection (*p* < 0.0001). FoxP3 expression levels (although not significant), followed the same trend as TGF-β1 in all cultures (Fig. 3, a-c). In PBMCs alone, TGF-β1 and FoxP3 expression levels decreased in response to *C. trachomatis* infection with or without the addition of azithromycin (Fig. 3, B), whereas in the presence of ECC1 cells, TGF-β1 and FoxP3 expression levels were upregulated in response to infection (Fig. 3, a and c).

### IFN-γ transcripts were not elevated in response to *C. trachomatis* infection in human PBMCs culture

Although IDO1 is known to be mainly induced by IFN-γ [11, 30, 40, 41], it has been previously shown to be induced by LPS in an IFN-γ-independent manner [31]. We therefore measured IFN-γ expression levels in response to infection in our in vitro model and found that in PBMCs alone, *C. trachomatis* infection did not affect IFN-γ expression levels, whereas IDO1 levels were significantly upregulated (Fig. 3, B). Interestingly, only when *C. trachomatis*-infected PBMCs were treated with azithromycin 20 h PI (+CT + AZ), IFN-γ transcript levels were upregulated in comparison to the control (*p* < 0.05) (Fig. 3, b; −CT –AZ). This suggests that IDO1 levels in the *C. trachomatis* infected cells were induced by a different mechanism other than IFN-γ. In ECC1 and PBMCs co-culture, IFN-γ levels were upregulated in response to *C. trachomatis* infection, however, not significantly.

### *C. trachomatis* infection of ECC1 cell line induced TGF-β1 and FoxP3 expression levels, but no IDO1 or IFN-γ transcripts were detectable

In ECC1 culture alone, there were no detectable IDO1 or IFN-γ expression levels in response to *C. trachomatis* infection (Fig. 3, a), or in any of the treatments. On the other hand, TGF-β1 and FoxP3 expression levels were detected in ECC1 culture during all conditions. Furthermore, TGF-β1 expression levels were significantly upregulated upon *C. trachomatis* infection, with or without azithromycin (*p* < 0.0001). This confirms that *C. trachomatis* stimulation of ECC1 alone can sufficiently trigger the expression of TGF-β1. Whereas ECC1 cells alone cannot express IDO1 in response to *C. trachomatis* infection, and may require a secondary stimulation by PBMCs.

## Discussion

As an intracellular bacterium, *Chlamydia* has developed mechanisms to avoid the immune response while successfully infecting its target host cell. Downregulation of the immune response by *Chlamydia* has been suggested to occur during infection [42–44], and perhaps is an additional contributing factor to the asymptomatic nature of the infection. Although IDO1 is induced during pathogen invasion and was proposed to have an antimicrobial activity via tryptophan depletion, overexpression of IDO1 was also shown to trigger an immune cascade, which eventually downregulates the pro-inflammatory response [19]. The cytokine IFN-γ is one of the main inducers of IDO1 [40], paradoxically, it was also associated with immune protection against *C. trachomatis* infections in several human studies [5–7].

**Fig. 4:**
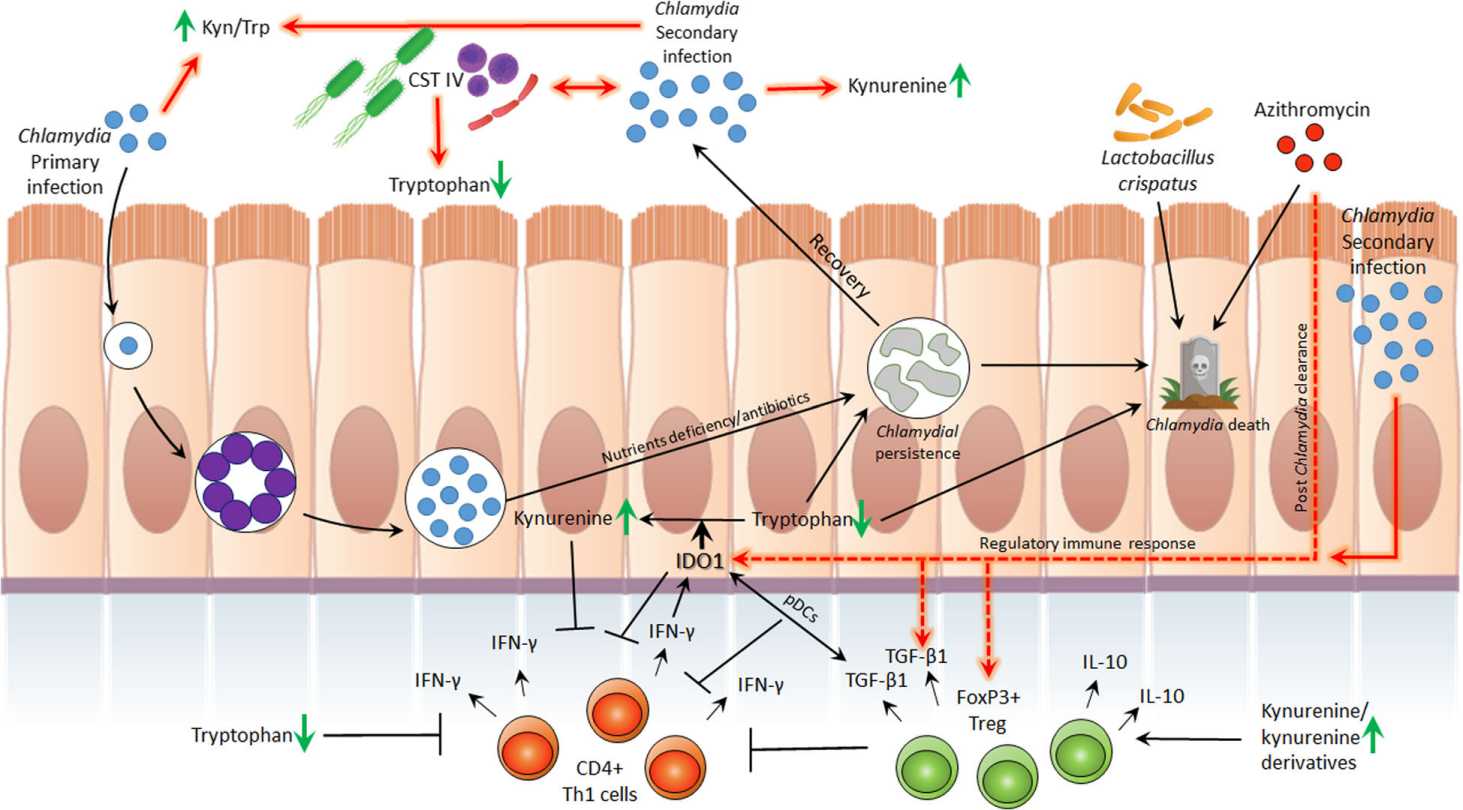
Immunological, bacterial and biochemical factors that are associated with initial or repeated *C. trachomatis* infections in women. In the figure, the chlamydial developmental cycle is described. Infection is initiated with the chlamydial EBs that convert into RBs, multiply and convert back to EBs. Then, the infectious progeny is released from the host cell to initiate an additional cycle. Upon infection, immune cells are recruited to the infected area, among them CD4+ expressing Th1 cells that produce IFN-γ. IFN-γ induces the production of IDO1 that catabolizes tryptophan into kynurenine, depleting the host tryptophan pools. This triggers the tryptophan auxotroph *Chlamydia* to enter its persistence form, or in severe tryptophan starvation, to its death. Vaginal tract microbiota has an important role in health and disease. Among these bacterial communities, CST IV was associated with current or previous *Chlamydia* infection, low tryptophan levels and high kynurenine/tryptophan ratios. On the other hand, vaginal *Lactobacillus crispatus* was shown to inhibit chlamydial growth. Initial and repeated *Chlamydia* infections were associated with high kynurenine/tryptophan ratios. Repeated *Chlamydia* infection was shown to be associated with high kynurenine levels. Although low tryptophan levels were found to inhibit *Chlamydia* in vitro and were associated with natural clearance in vivo, tryptophan depletion is also related to the inhibition of Th1 immunity. Kynurenine and IDO1 are also known to inhibit T cells and local immunity. IDO1 and TGF-β1 are known to synergistically activate tolerogenic effect in pDCs. Azithromycin was shown to be effective in killing *Chlamydia*, however, also eliciting an anti-inflammatory response. *Chlamydia* infection clearance post azithromycin treatment in women was found to elicit IDO1, TGF-β1 and FoxP3 regulatory immune response. Repeated *Chlamydia* infection in women had similar effects on the expression levels of these genes, and might have been triggered by high kynurenine/tryptophan ratios. *Chlamydia* infection in in vitro ECC1 and PBMCs co-culture was found to elicit IDO1, TGF-β1 and FoxP3 as well

*C. trachomatis* inhibition due to IDO1-induced tryptophan depletion has been a major hypothesis in the field [8, 9], and here we sought to identify the IDO1 response in infected women and to evaluate its association with the immune-regulatory response. Despite the small number of patients we found that *C. trachomatis* infected women, either single or repeated infection, had significantly higher IDO1 expression levels. This result, along with previously reported data by Ziklo et al., (2018), of significantly higher kyn/trp ratios in the same cohort of women, suggest that tryptophan metabolism via IDO1 is indeed active in the genital tract during *C. trachomatis* infection in women (Fig. 4). Surprisingly, we also observed a significant upregulation in IDO1 expression levels in women who cleared their infection post antibiotic treatment (PAT; Fig. 1, a and Additional file 4, a). These women however, were reported to have low kyn/trp ratios [17]. In addition, we have previously reported that these women who had cleared their infection had a variety of cervicovaginal microbial communities, comprised of community state types (CSTs) I (45.5% of the women in PAT group), which is dominated by *Lactobacillus crispatus*, CST III (36.5%), which is dominated by *L. iners*, and CST IV (18%), which comprised of diverse bacterial species and considered as dysbiosis (Additional file 2). Although previous literature has shown that gut *Lactobacillus spp.*, can reduce host IDO immune response and promote mucosal protection and colonization resistance to pathogens via IL-22 [45–48], in this study we did not observe an effect of cervicovaginal microbiota CST on IDO1 expression levels. Nevertheless, we have previously reported that in the same cohort, women who had CST IV had significantly lower levels of tryptophan in their vaginal secretions [17] (Fig. 4). Because IDO1 levels in women who cleared their infection post antibiotic treatment (PAT) did not correlate with the low kyn/trp ratios, we hypothesized that perhaps IDO1 is upregulated by a different mechanism, such as the long-term tolerogenic effect found in pDCs, in response to a synergistic effect with TGF-β1, which is independent of its enzymatic activity [29]. In addition, DCs were reported in a few studies to have an important role regulating the host immune response to infection with several *Chlamydia spp.* [42, 44, 49, 50]*.* We found that similar to IDO1 expression levels, women with repeated *C. trachomatis* infections had significantly higher TGF-β1 levels, as well as in women who cleared their infection post antibiotic treatment (Fig. 1, B and Additional file 4, B). However, in contrast to IDO1, women with a single *C. trachomatis* infection had lower TGF-β1 expression levels in comparison to *C. trachomatis* negative women (not significant). When measuring FoxP3 expression levels, only women post antibiotic treatment had significantly higher expression levels.

These data suggest that IDO1 might have a different role when being expressed during different *C. trachomatis* infection-statuses. During antigen presentation to the immune system, hence, an active *C. trachomatis* infection, either single or repeated, tryptophan catabolism to kynurenine is active, as previously reported [17], correlating with the upregulation of IDO1. However, anti-inflammatory cytokine TGF-β1 is highly expressed only during repeated infection and secondary immune recognition. In case of infection clearance post antibiotic treatment, without antigen presentation, all three genes measured were significantly higher, even without noticeable tryptophan catabolism to kynurenine [17]. These data correlate with a previous study in trachoma that showed sustained FoxP3 expression levels in the absence of an active *C. trachomatis* infection [16]. Significant expression levels of TGF-β1 only during secondary antigen presentation might suggest its role as a memory immune response to *C. trachomatis* infections. The increased expression levels of IDO1, TGF-β1 and FoxP3, in women who cleared their infection, may suggest that during the combined effect of highly expressed levels of IDO1 and TGF-β1, this induces the expression levels of FoxP3, an indicator of regulatory immune response [29]. In addition, the use of antibiotics have been previously shown to dampen the immune response and promote tolerance [51, 52], and perhaps in this case, azithromycin treatment directly contributed to the upregulation of TGF-β1 and FoxP3 expression levels.

In our in vitro ECC1 and PBMCs co-culture model we were able to show that IDO1 and TGF-β1 expression levels were significantly upregulated in response to *C. trachomatis* infection. Surprisingly, while in the *C. trachomatis* infected co-culture immune-regulatory TGF-β1 and FoxP3 genes were upregulated, in PBMCs alone, expression levels of these genes were downregulated. This downregulation was significant for TGF-β1 in response to *C. trachomatis* infection or azithromycin treatment alone in PBMCs. Here, we show that the infectivity of *C. trachomatis* in PBMCs yielded significantly reduced IFUs/ml, consistent with previous literature indicating that *C. trachomatis* can infect DCs, monocytes and macrophages [53, 54]. Although the infectivity in PBMCs is reduced in comparison to epithelial cells, introducing this pathogen to a naïve PBMCs culture was previously shown to elicit an immune response, specifically downregulation of the pro-inflammatory response via IL-10 [55]. This emphasizes the cross talk between ECC1 cells and the PBMCs when cultured together, in which TGF-β1 and FoxP3 switched to upregulated expression levels upon chlamydial infection. This might suggest that the PBMCs are programed to act in a pro-inflammatory manner in response to a bacterial infection, however, a signal from the epithelial cells promote regulation, in order to protect the tissue from damage. This effect was also demonstrated in ECC1 culture alone, where TGF-β1 and FoxP3 were upregulated in response to *C. trachomatis* infection (significant effect was shown for TGF-β1 but not in FoxP3). FoxP3 is a key transcription factor previously thought to be expressed mainly in Tregs and is used to assess immune-regulatory function of the host [56]. It was previously shown to be also expressed by several epithelial cell types such as thymic and breast cells [57]. Here, we show that FoxP3 can also be expressed by ECC1 cell line and is upregulated in response to *C. trachomatis* infection. Although IFN-γ is known to be the main inducer of IDO1 expression levels, in PBMCs culture alone we found that the significant upregulation of IDO1 was not accompanied by elevated IFN-γ. In fact, *C. trachomatis* infection did not induce any upregulation in IFN-γ expression levels when compared to the control (−CT -AZ). This suggests that IFN-γ was not solely responsible for the significant induction of IDO1 levels in PBMCs and there is another mechanism, perhaps the stimulation of TLRs by chlamydial particles and innate recognition, which triggered IDO1 expression. Moreover, these in vitro data also correlate with the discordancy in low IFN-γ levels in vaginal secretions of women from the same cohort, with either single or repeated *C. trachomatis* infection, and the high kyn/trp ratios [17], along with the high IDO1 expression levels measured in this study.

Although the PBMCs used in this study were sourced from a single donor, the in vitro model was designed in order to control for variations between donors’ immune response. All treatments were compared with *C. trachomatis* negative culture, while the effect of the PBMCs and ECC1 co-culture was measured by comparing it to single ECC1 or PBMCs culture. Furthermore, the donor was a healthy woman who had no previous *C. trachomatis* infections.

While previous studies have shown that IDO can be expressed by some mucosal epithelial and tumor cells [58, 59], we found that in ECC1 culture alone, IDO1 expression levels were not detectable in response to *C. trachomatis*. Although IFN-γ expression levels were not upregulated in response to *C. trachomatis* infection in PBMCs alone, the addition of PBMCs to the ECC1 culture may have provided additional secreted cytokines, other than IFN-γ, that elicit the immune response cascade and subsequent induction of IDO1. Recently, a study by Jordan et al. (2017), has shown that TNF-α was the predominant cytokine elicited in stimulated PBMCs from *C. trachomatis* infected patients, rather than IFN-γ [60]. In addition, TNF-α was also shown to increase IFN-γ-induced IDO1 levels [31]. Perhaps, the subtle IFN-γ secreted by the PBMCs, along with other secreted cytokines were responsible for the significant IDO1 expression levels in PBMCs culture alone and in the co-culture. Nevertheless, the in vivo conditions of cervical epithelial cells infected with *C. trachomatis*, triggering the infiltration of immune cells to the infected area, are far more complex than in vitro co-culture environment. Despite these limitations, we were able to show that there is cross talk between the infected epithelial cell line and the PBMCs in response to *C. trachomatis* infection, while this response is comprised of a significant increase in IDO1 and TGF-β1 expression levels. In addition, these in vitro results correlate with the significantly increased expression levels of IDO1 and TGF-β1 in vaginal swab samples from women who are repeatedly infected with *C. trachomatis.*

## Conclusions

Our results add weight to the available published data of the regulatory immune response to *C. trachomatis* vaginal tract infections in women, in which differs in case of a single versus repeated infection. In addition, we were able to show that *C. trachomatis* infection in PBMCs did not elicit IFN-γ response, in spite the significant expression levels of IDO1. These preliminary data may prompt future research into a different key cytokine to elicit IDO1 response and subsequent tryptophan depletion, in order to eliminate *C. trachomatis* infection in vitro. In addition, this underscore IDO1 and TGF-β1 as a memory immune response to *C. trachomatis* and their role in driving immune regulation. Consequently, our research provides evidence to include measurements of these immune factors in future vaccine studies.

## Additional files

Additional file 1: Patients’ information summary. (DOCX 12 kb)

Additional file 2: Detailed participant’s information. (DOCX 14 kb)

Additional file 3: Microscopic image of *Chlamydia*-infected ECC1 host cells. Monolayer of ECC1 alone or a co-culture of ECC1 and *Chlamydia*-negative female donor PBMCs were infected with *C. trachomatis* at MOI of 0.1 and were harvested at 44 h PI. Cultures were either treated with azithromycin at 20 h PI, or not. Cells and chlamydial inclusions were stained in Chlamydia CEL LPS (Cellabs, Australia). Figure show (A) ECC1 cells infected with *C. trachomatis*. (B) ECC1 cells infected with *C. trachomatis* and treated with azithromycin. (C) Co-culture of ECC1 and PBMCs infected with *C. trachomatis*. (D) Co-culture of ECC1 and PBMCs infected with *C. trachomatis* and treated with azithromycin. (TIF 35745 kb)

Additional file 4: Longitudinal IDO1 and TGF-β1 expression levels from vaginal swab samples of women from initial *Chlamydia* infection and post antibiotic treatment in follow-up visits. Expression levels of (A) IDO1 and (B) TGF-β1 were measured from vaginal swab samples of women who were *Chlamydia* positive at first visit (time point 0), cleared their infection after azithromycin treatment, and were invited for follow up visits (1–4 months post infection clearance). Results are presented as 2^(− ΔCT)^. (TIF 49 kb)

## Abbreviations

FoxP3: Forkhead box P3
IDO: Indoleamine-2,3 dioxygenase
IFN-γ: Interferon-gamma
PBMCs: Peripheral blood mononuclear cells
pDCs: Plasmacytoid dendritic cells
TGF-β: Transforming growth factor β
Treg: Regulatory T cells
