# Supplementary material for: High expression of IDO1 and TGF-β1 during recurrence and post infection clearance with Chlamydia trachomatis, are independent of host IFN-γ response
Source: BMC Infect Dis. 2019 Mar 4;19:218. doi: 10.1186/s12879-019-3843-4 (PMC6398247; doi:10.1186/s12879-019-3843-4)
Supplement: Supplementary file 1 — Patients’ information summary. (DOCX 12 kb) [file 12879_2019_3843_MOESM1_ESM.docx]

**Additional file 1** Patients' information summary. Table

| Group | Number of patients | *Chlamydia* status | Months post antibiotic treatment |
| --- | --- | --- | --- |
| CT-N | 7 | Negative | N.A. |
| CT-P | 8 | Positive | N.A. |
| PAT | 11 | Negative | 1-4 |
| CT-RP | 3 | Positive | >3 |
