# Supplementary material for: High expression of IDO1 and TGF-β1 during recurrence and post infection clearance with Chlamydia trachomatis, are independent of host IFN-γ response
Source: BMC Infect Dis. 2019 Mar 4;19:218. doi: 10.1186/s12879-019-3843-4 (PMC6398247; doi:10.1186/s12879-019-3843-4)
Supplement: Supplementary file 2 — Detailed participant’s information. (DOCX 14 kb) [file 12879_2019_3843_MOESM2_ESM.docx]

**Additional file 2** Detailed participant’s information

| **Patient** | **Group** | **Community state type (CST)** | **Presence of clue cells** | **Nugent score** | **pH** | **Age** |
| --- | --- | --- | --- | --- | --- | --- |
| 102 | CT-N | III | - | 2 | 4.5 | 25 |
| 103 | CT-N | I | - | 0 | 4.0 | 27 |
| 108 | CT-N | I | - | 1 | 3.5 | 47 |
| 111 | CT-N | I | + | 6 | 4.5 | 22 |
| 106 | CT-N | III | - | 1 | 4.5 | 27 |
| 109 | CT-N | III | - | 2 | 4.5 | 48 |
| 110 | CT-N | IV | + | 9 | 5.0 | 24 |
| 201 | CT-P | III | - | 3 | 4.5 | 21 |
| 207 | CT-P | I | - | 0 | 4.0 | 32 |
| 209 | CT-P | III | - | 2 | 4.5 | 27 |
| 211 | CT-P | III | - | 2 | 4.0 | 19 |
| 214 | CT-P | I | + | 6 | 4.5 | 19 |
| 217 | CT-P | III | + | 6 | 4.5 | 29 |
| 219 | CT-P | IV | + | 9 | 6.5 | 35 |
| 224 | CT-P | III | - | 1 | 4.0 | 26 |
| 203 | PAT | I | + | 6 | 4.0 | 21 |
| 204 | PAT | I | + | 6 | 4.5 | 32 |
| 205 | PAT | III | + | 8 | 5.0 | 27 |
| 206 | PAT | I | - | 0 | 4.0 | 24 |
| 212 | PAT | IV | + | 6 | 4.5 | 27 |
| 215 | PAT | I | - | 3 | 4.5 | 32 |
| 216 | PAT | III | + | 7 | 4.0 | 21 |
| 220 | PAT | III | - | 0 | 4.0 | 35 |
| 221 | PAT | III | - | 0 | 4.0 | 26 |
| 250 | PAT | I | + | 5 | 4.0 | 19 |
| 312 | PAT | IV | + | 9 | 5.0 | 29 |
| 213 | CT-RP | III | - | 2 | 4.0 | 24 |
| 306 | CT-RP | IV | + | 10 | 5.5 | 22 |
| 319 | CT-RP | III | + | 5 | 4.5 | 26 |
