# Supplementary figures and images for: High expression of IDO1 and TGF-β1 during recurrence and post infection clearance with Chlamydia trachomatis, are independent of host IFN-γ response
Source: BMC Infect Dis. 2019 Mar 4;19:218. doi: 10.1186/s12879-019-3843-4 (PMC6398247; doi:10.1186/s12879-019-3843-4)

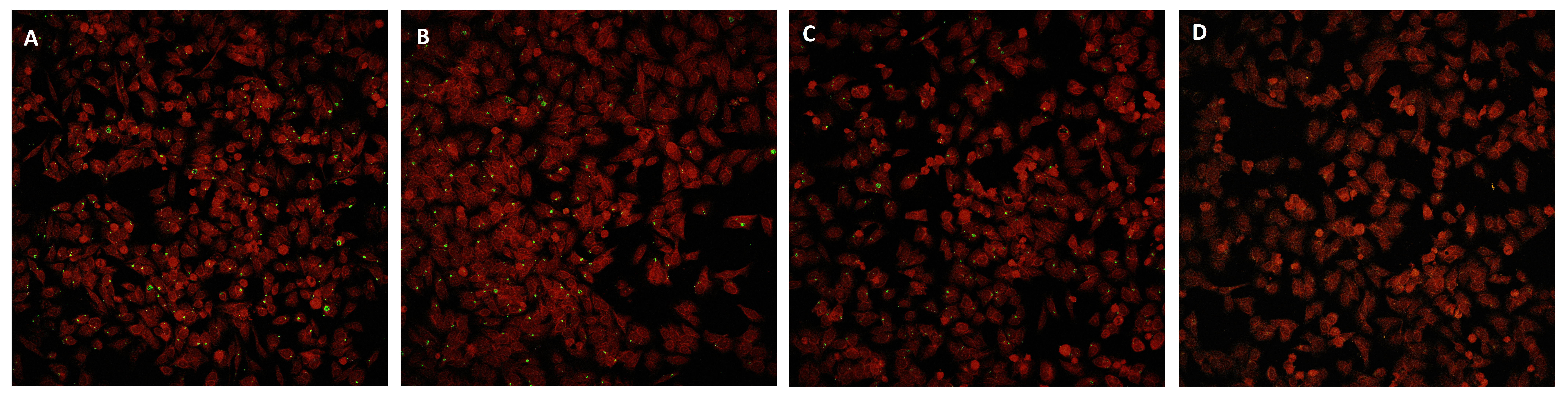

Supplement: Supplementary file 3 — Microscopic image of Chlamydia-infected ECC1 host cells. Monolayer of ECC1 alone or a co-culture of ECC1 and Chlamydia-negative female donor PBMCs were infected with C. trachomatis at MOI of 0.1 and were harvested at 44 h PI. Cultures were either treated with azithromycin at 20 h PI, or not. Cells and chlamydial inclusions were stained in Chlamydia CEL LPS (Cellabs, Australia). Figure show (A) ECC1 cells infected with C. trachomatis. (B) ECC1 cells infected with C. trachomatis and treated with azithromycin. (C) Co-culture of ECC1 and PBMCs infected with C. trachomatis. (D) Co-culture of ECC1 and PBMCs infected with C. trachomatis and treated with azithromycin. (TIF 35745 kb) [file 12879_2019_3843_MOESM3_ESM.tif]

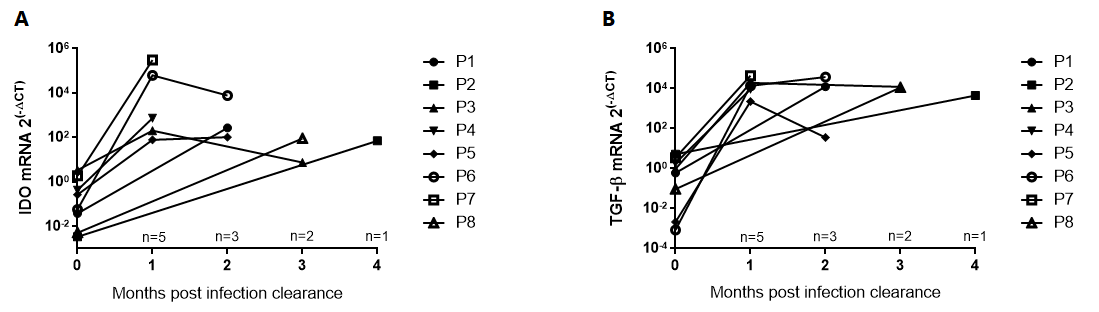

Supplement: Supplementary file 4 — Longitudinal IDO1 and TGF-β1 expression levels from vaginal swab samples of women from initial Chlamydia infection and post antibiotic treatment in follow-up visits. Expression levels of (A) IDO1 and (B) TGF-β1 were measured from vaginal swab samples of women who were Chlamydia positive at first visit (time point 0), cleared their infection after azithromycin treatment, and were invited for follow up visits (1–4 months post infection clearance). Results are presented as 2(− ΔCT). (TIF 49 kb) [file 12879_2019_3843_MOESM4_ESM.tif]
